# Statistical Optimization of Conditions for Decolorization of Synthetic Dyes by *Cordyceps militaris* MTCC 3936 Using RSM

**DOI:** 10.1155/2015/536745

**Published:** 2015-02-04

**Authors:** Baljinder Kaur, Balvir Kumar, Neena Garg, Navneet Kaur

**Affiliations:** Department of Biotechnology, Punjabi University, Patiala 147002, India

## Abstract

In the present study, the biobleaching potential of white rot fungus *Cordyceps militaris* MTCC3936 was investigated. For preliminary screening, decolorization properties of *C. militaris* were comparatively studied using whole cells in agar-based and liquid culture systems. Preliminary investigation in liquid culture systems revealed 100% decolorization achieved within 3 days of incubation for reactive yellow 18, 6 days for reactive red 31, 7 days for reactive black 8, and 11 days for reactive green 19 and reactive red 74. RSM was further used to study the effect of three independent variables such as pH, incubation time, and concentration of dye on decolorization properties of cell free supernatant of *C. militaris*. RSM based statistical analysis revealed that dye decolorization by cell free supernatants of *C. militaris* is more efficient than whole cell based system. The optimized conditions for decolorization of synthetic dyes were identified as dye concentration of 300 ppm, incubation time of 48 h, and optimal pH value as 5.5, except for reactive red 31 (for which the model was nonsignificant). The maximum dye decolorizations achieved under optimized conditions for reactive yellow 18, reactive green 19, reactive red 74, and reactive black 8 were 73.07, 65.36, 55.37, and 68.59%, respectively.

## 1. Introduction

Environmental pollution is regarded as one of the major hazards of the world due to rapid industrialization [1]. Synthetic dyes are extensively used in various industries including textile, paper, printing, cosmetics, and pharmaceuticals [2]. Synthetic dyes have complex aromatic molecular structures which make them more stable and difficult to biodegrade [3]. The three most common groups of synthetic dyes include azo, anthraquinone, and phthalocyanine. Most of them are toxic and carcinogenic in nature [4]. These dyes also affect the photosynthetic activity of hydrophytes significantly by lowering the light penetration in water and breakdown products may be toxic to aquatic organisms [5, 6].

For the treatment of industrial textile dye containing effluents, various physical and chemical treatment methods including adsorption, coagulation, precipitation, filtration, and oxidation have been extensively used. These processes generate a significant amount of sludge which results in secondary pollution due to excessive usage of chemicals and reagents. Moreover, the treatment cost is also high. Therefore, alternative means of dye decolorization are necessary to develop, such as bioremediation which is an environmentally friendly and publicly acceptable treatment process [5]. Organic compounds are completely converted into water and carbon dioxide by several microorganisms [7]. Various bacteria and fungi are used in dye decolorization and in many cases adsorption of dyes on the microbial cell surface is the primary mechanism for decolorization of the dyes [8].

White rot fungi are key regulators of the global C-cycle and decompose synthetic dyes under aerobic conditions. Their lignin modifying enzymes (LME) are mainly oxidoreductases, that is, manganese peroxidases (MnP), E.C. 1.11.1.13, lignin peroxidases (LiP), E.C. 1.11.1.14, and laccases (Lac), E.C. 1.10.3.2, which are directly involved not only in the degradation of lignin in their natural lignocellulosic substrates but also in the degradation of various xenobiotic compounds including dyes [9]. Due to its extracellular and nonspecific nature, free radical based ligninolytic system of white rot fungi can completely eliminate a variety of pollutants and xenobiotics such as polychlorinated biphenyls (PCB), polycyclic aromatic compounds (PAH), pesticides, synthetic dyes, and industrial dyes and produces nontoxic compounds [10–12]. However, there is lack of scientific evidence reporting dye decolorization ability of the* C. militaris* and its use in bioremediation of textile industry effluents.

Response surface methodology (RSM) is an efficient experimental strategy to determine optimal conditions for a multivariable system rather than optimization by the conventional method which involves changing one independent variable while keeping the other factors constant. Conventional methods are also time-consuming and incapable of detecting the true optimum, especially the absence of interactions among factors and in defining the effect of the independent variables, alone or in combination, on the processes [13].

The primary goal of the study was statistical optimization of dye decolorization by cell-free supernatant of* Cordyceps militaris* MTCC 3936 using response surface methodology. Preliminary experiments were conducted in agar based as well as liquid culture systems to identify dye tolerance capacity of the fungus which was estimated using wet weight analysis and percentage dye decolorization as prominent responses.

## 2. Materials and Methods

### 2.1. Materials

Synthetic textile dyes, namely, reactive yellow 18, reactive green 19, reactive red 31, reactive red 74, and reactive black 8, used in the study were obtained from a textile industry of Punjab, India. All other chemicals, used in the study, were procured from HiMedia and were of analytical grade.

### 2.2. Microorganism and Cultural Conditions

Pure culture of fungus* Cordyceps militaris* MTCC 3936 was procured from IMTECH, Chandigarh. The culture was maintained on cornmeal agar slants at 4°C and subcultured at required intervals. Cornmeal agar medium consisted of 30 g cornmeal and 20 g agar. Inoculum was prepared by transferring a loopful of fungal culture from slant to 100 mL sterilized cornmeal medium and incubated at 20°C for 7 days under stationary conditions.

### 2.3. Screening of the Dyes for *λ*_max⁡_

The stock solutions of dyes were prepared at different concentrations ranging from 50 to 300 ppm in distilled water. *λ*_max⁡_ screening of these dye solutions was carried out at a wavelength range of 320 nm to 760 nm using UV-VIS spectrophotometer (Thermo-Spectronic).

### 2.4. Assay of Dye Decolorization on Synthetic Minimal Agar Media

Freshly prepared inoculum of* C. militaris* MTCC 3936 was spotted in the centre of Petri plates containing synthetic minimal agar medium (containing glucose 10 g/L, ammonium sulphate 5 g/L, potassium phosphate monobasic 5 g/L, magnesium hepta sulphate 1 g/L, and agar 20 g/L and pH 6.5 ± 0.2) supplemented with reactive dyes at various concentrations from 200 to 1000 ppm. Petri plates were incubated at 20°C for 15 days and were manually analyzed for the dye decolorization regularly after every 24 h intervals [14].

### 2.5. Preliminary Screening of Dye Decolorization Properties of* C. militaris* MTCC 3936 Using Liquid Culture Systems

#### 2.5.1. Dye Decolorization Capacity

Synthetic minimal medium was supplemented with dyes at different concentrations ranging from 50 to 250 ppm. 1% (w/v) freshly grown inoculum of* C. militaris* MTCC 3936 was used for inoculation. After inoculation, flasks were kept at 20°C for 15 days. Controls were prepared containing minimal media and respective dye and synthetic minimal media without dye were taken as blank. 2 mL of the solution was withdrawn from each flask after every 24 h interval up to 15 days. Samples were centrifuged at 10,000 rpm for 10 min to settle down the culture. Supernatants were collected separately and absorbance of dyes was measured at the *λ*_max⁡_ already determined [15]. % dye decolorization was calculated as

(1) %  dye  decolorization =Initial  absorbance−Final  absorbanceInitial  absorbance×100.

#### 2.5.2. Wet Weight Analysis

For wet weight analysis, fungal biomass was grown in synthetic minimal media supplemented with different dyes (50–250 ppm) at 20°C for 15 days. The liquid media were filtered through Whatman's filter paper number 1 and the wet biomass on the filter paper was weighed.

### 2.6. Preparation of Culture Filtrate for Assaying Dye Decolorization

The culture of* Cordyceps militaris* was grown in respective media and incubated at 20°C for 10 days. The cell-free supernatant of fungal culture was separated by centrifugation at 10,000 rpm for 10 min and used for assaying dye decolorization.

### 2.7. Optimization of Conditions for Dye Decolorization Using Response Surface Methodology (RSM)

Experimental design was made by using Design Expert software version 8.0.7.1. Centre composite rotatable design (CCRD) was used to optimize dye decolorization as a function of three variable factors, that is, dye concentration (ppm), incubation time (h), and pH of the medium, and to study the interactions among selected variables in each experiment. The experimental plan consisted of 20 sets. Synthetic minimal media containing different concentrations of dyes were prepared as described in CCRD runs (Table 3). The cell-free supernatant from freshly grown culture of* C. militaris* was used in the assay which was added at a level of 300 *μ*L per 15 mL of media.

## 3. Results

### 3.1. Assay of Dye Decolorization on Synthetic Minimal Agar Media

**Figure 1:**
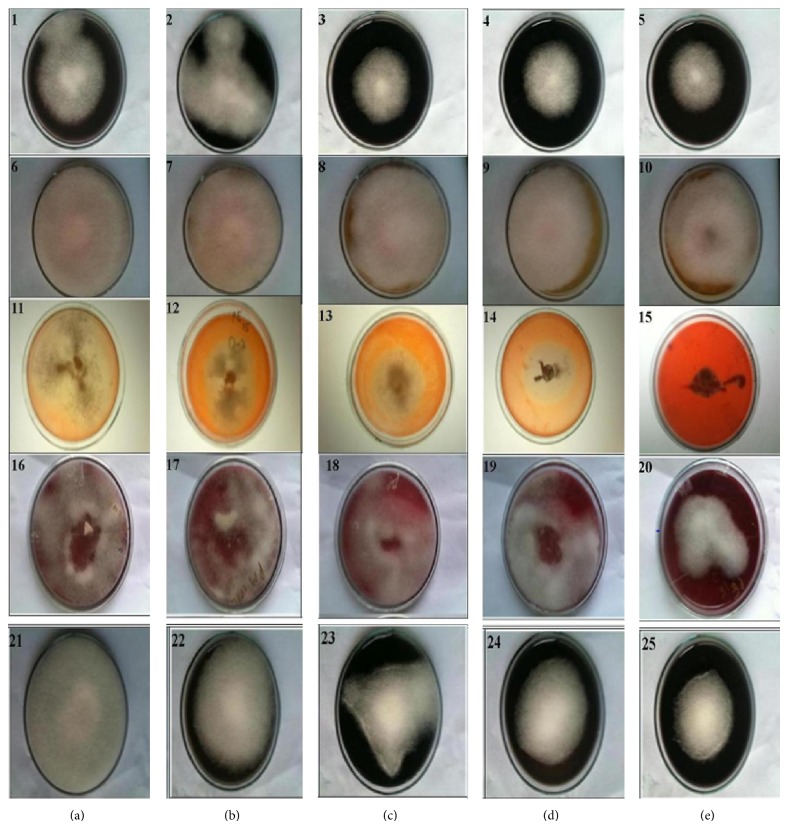
Assay plates showing dye decolorization by* C. militaris* MTCC 3936 after 10 days of growth, where (a) to (e) represent dye concentrations of 200 to 1000 ppm and assay plates 1–5 depict reactive green 19, 6–10 reactive yellow 19, 11–15 reactive red 31, 16–20 reactive red 74, and 21–25 reactive black 8.

In case of reactive red 74, dye clearance zones were observed after 3 days of incubation as compared to other reactive dyes. The growth of the fungi was inversely linked to dye concentration as lesser growth was observed in media containing higher concentration of dye and vice versa, which indicates toxicity of the dye towards* Cordyceps militaris*. However, a positive correlation was found between the fungal growth rate and its decolorization ability.  Figure 1 shows growth pattern of the fungus on minimal agar media supplemented with various synthetic dyes where growth of the fungus was retarded as the concentration of the dye increased from 200 to 1000 ppm. Results indicate poor degradation ability of the fungus in agar based dye decolorization assay, which might be due the higher concentrations of dyes used (from 0.02 to 0.1% w/v) or due to the fact that fungus requires longer incubation periods for complete dye removal. So, further assessment of dye decolorization was performed in liquid culture systems where dye is more accessible to fungal enzymes.

### 3.2. Preliminary Screening of Dye Decolorization Properties of* C. militaris* MTCC 3936 Using Liquid Culture Systems

**Figure 2:**
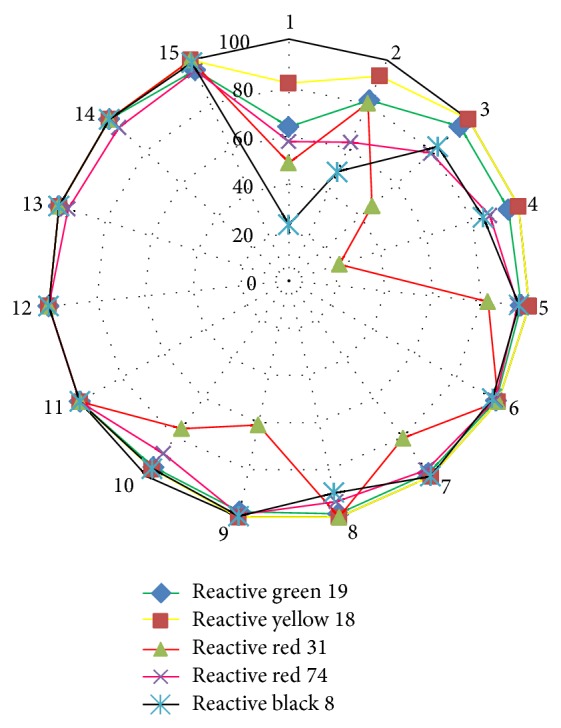
Comparative analysis of dye decolorization properties of* C. militaris* MTCC 3936 in liquid cultures.

**Figure 3:**
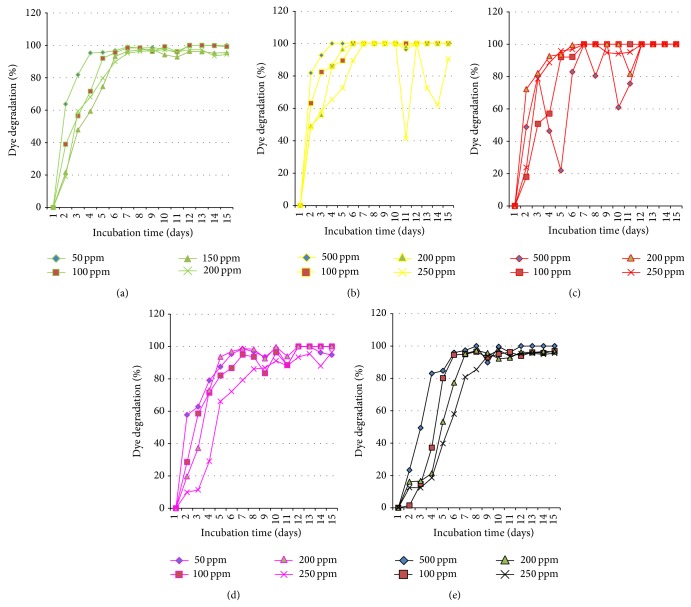
Graphical correlation of dye degradation capacity with respect to incubation time in* C. militaris* MTCC 3936. (a) Reactive green 19; (b) reactive yellow 18; (c) reactive red 31; (d) reactive red 74; (e) reactive black 8.

Preliminary investigation in liquid culture systems revealed 100% dye decolorization achieved within 3 days of incubation for reactive yellow 18, 6 days for reactive red 31, 7 days for reactive black 8, and 11 days for reactive green 19 and reactive red 74 when* C. militaris* MTCC 3936 growth medium was supplemented with dyes at 50 ppm strength (Table 1). Longer time periods were required for complete removal of dyes added at higher concentrations. As observed in Figures 2 and 3, fungal biomass shows frequent leaching of the dye stuff into the liquid medium which is especially prominent in case of dye reactive red 31. This behavior of the fungus further complicates the assay of decolorization in liquid medium and might be responsible for the generation of nonsignificant model for reactive red 31 dye during CCRD analysis. For other dyes, 100% decolorization was achieved within 6 to 11 days of incubation where leaching of the dye from fungal hyphae is not a very prominent phenomenon.

The dyes were removed rapidly from the liquid culture by absorption process and later were degraded by the enzymes synthesized by fungal strain. Complete adsorption of dyes to fungal biomass was observed after 15 days of incubation as no residual dye stuff was left in the medium except for dyes reactive green 19 and reactive black 8 at concentrations more than 200 ppm (Table 1 and Figure 3).

Wet weight analysis indicated inhibition of fungal growth as a function of dye concentration as the more the dye concentration (increases from 50 to 250 ppm), the more its toxicity towards fungal biomass (Table 1). Reactive red 31 is the most toxic dye as it showed maximum growth inhibition and reactive yellow 18 is the least toxic dye studied. Information related to their molecular weights as well as structure is not available in the literature, so, it is very difficult to correlate our findings with the molecular features of the synthetic dyes. Earlier, Sani and Banerjee [16] indicated that dyes with simple structures and low molecular weights have higher rates of dye decolorization, whereas dye decolorization was more difficult with highly substituted and high molecular weight dyes.

### 3.3. Optimization of Conditions for Dye Decolorization of Different Dyes Using Response Surface Methodology (RSM)

RSM based CCRD model was used to optimize variable factors that affect dye decolorization properties of cell-free supernatant containing crude enzyme mixture from* C. militaris* cultures. The maximum dye decolorization was achieved at dye concentration of 300 ppm, incubation time of 48 h, and pH of 5.5, except for reactive red 31 (for which the model turned to be nonsignificant). Under optimized conditions, reactive yellow 18, reactive green 19, reactive red 74, and reactive black 8 were decolorized to 73.07, 65.36, 55.37, and 68.59%, respectively, after 48 hours of incubation at 20°C.

From Table 2, it was concluded that the maximum experimental response as dye decolorization was 73.07% in case of reactive black 8, whereas the predicted value of the response was 72%. There was dye decolorization shown in other dyes also in order reactive yellow 18 (73.07%) > reactive black 8 (68.59%), reactive green 19 (65.36%) > reactive red 74 (55.37%) > reactive red 31 (49.11%). Results indicate a strong correlation between experimental responses and predicted responses that also depicts significance of the results obtained. The optimal conditions for dye decolorization by the fungal strain* C. militaris* MTCC 3936 have been predicted as pH 5.5, concentration 300 ppm, and incubation time 48 h in case of all the dyes.

**Figure 4:**
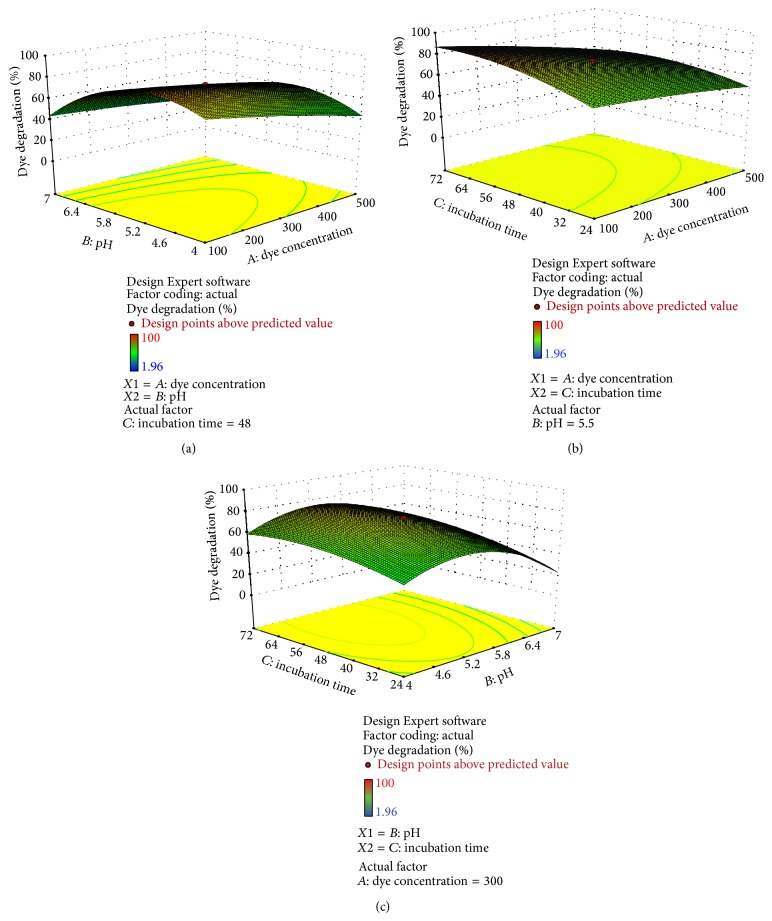
3D response surface plots showing dye decolorization of reactive yellow 18 as a function of (a)* AB*: pH and dye concentration; (b)* AC*: incubation time and dye concentration, and (c)* BC*: incubation time and pH.

**Figure 5:**
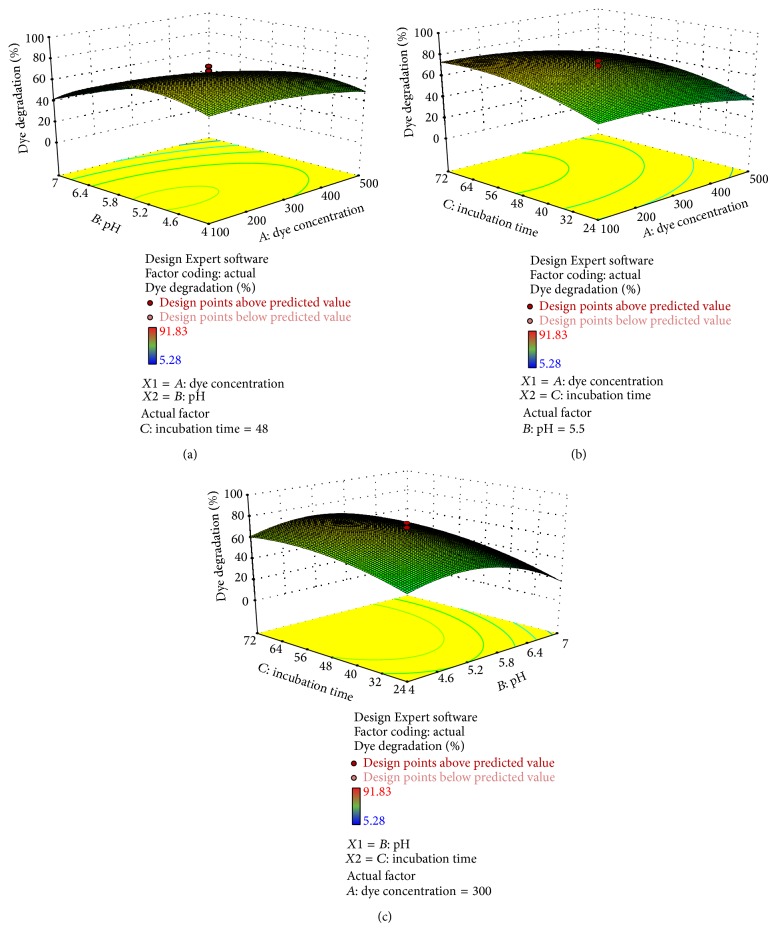
3D response surface plots showing dye decolorization of reactive green 19 as a function of (a)* AB*: pH and dye concentration; (b)* AC*: incubation time and dye concentration, and (c)* BC*: incubation time and pH.

**Figure 6:**
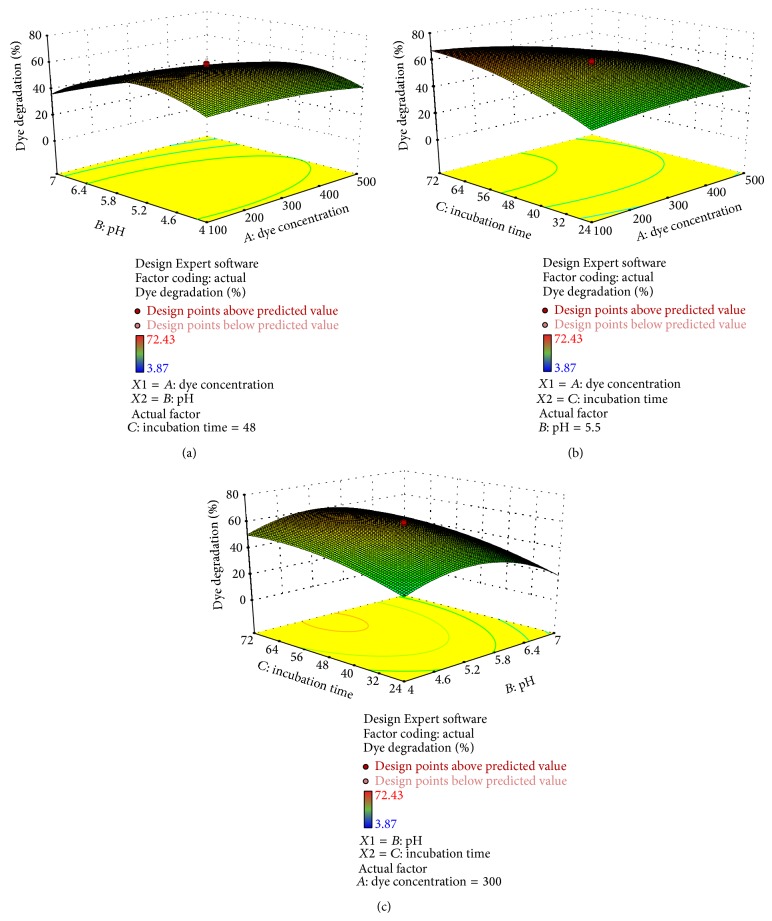
3D response surface plots showing dye decolorization of reactive red 74 as a function of (a)* AB*: pH and dye concentration; (b)* AC*: incubation time and dye concentration, and (c)* BC*: incubation time and pH.

**Figure 7:**
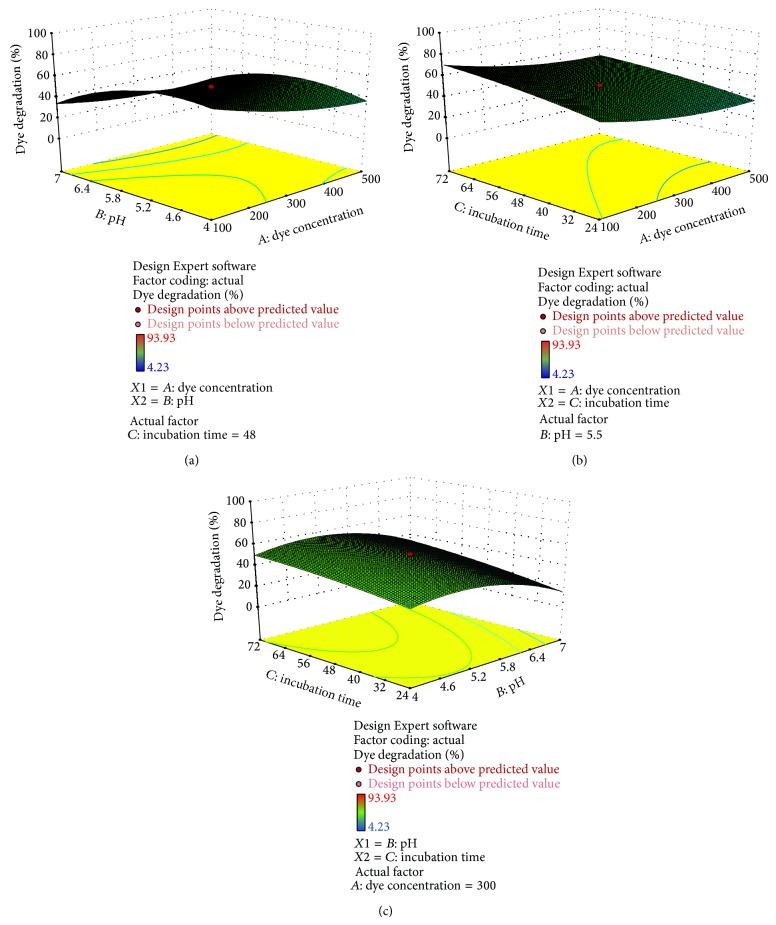
Response surface plots showing dye decolorization of reactive red 31 as a function of (a)* AB*: pH and dye concentration; (b)* AC*: incubation time and dye concentration, and (c)* BC*: incubation time and pH.

**Figure 8:**
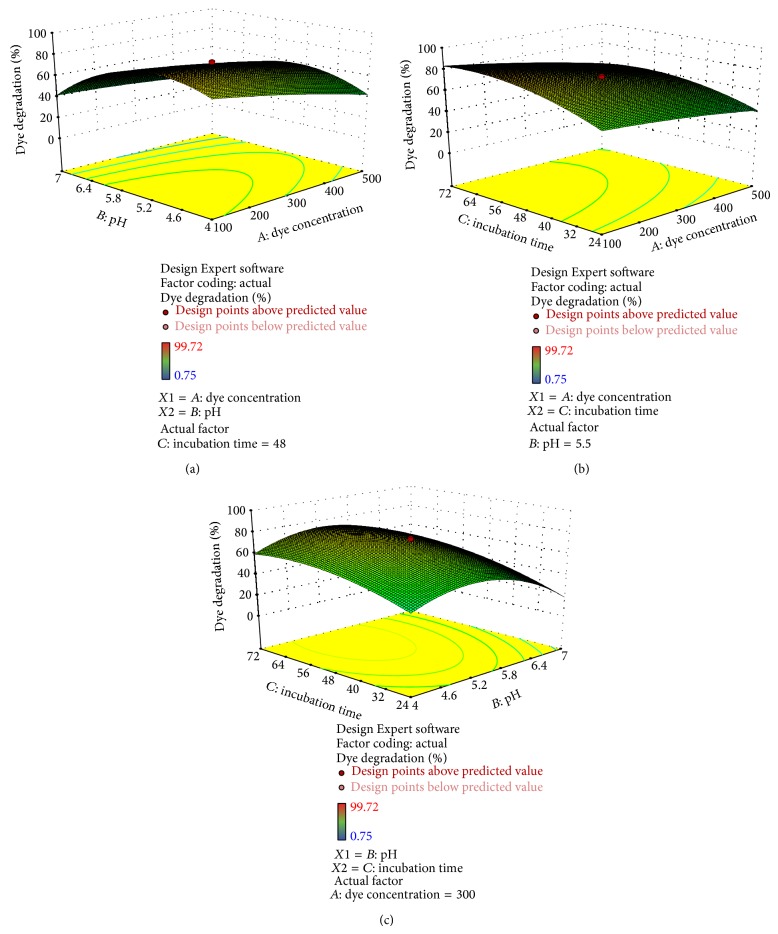
Response surface plots showing dye decolorization of reactive black 8 as a function of (a)* AB*: pH and dye concentration; (b)* AC*: incubation time and dye concentration, and (c)* BC*: incubation time and pH.

Figures 4, 5, 6, 7, and 8 represent experimental dye decolorization responses of* C. militaris* MTCC 3936 as 3D surface plots. Figures 4(a), 5(a), 6(a), and 8(a) show that pH and incubation time have nearly similar effects on the % dye decolorization. At a particular point, the % dye decolorization increases with increase in the incubation time up to the suggested optimal value which falls afterwards, but in comparison to other two variables, dye concentration has negligible effect on the observed response which is very clear from Figures 5, 6, 7, and 8; Table 3. Figures 4(c), 5(c), 6(c), and 8(c) show that pH greatly influences dye decolorization rate, while incubation time has negligible effect on the decolorization of reactive dyes.

The dye decolorization responses obtained using cell-free supernatants of* C. militaris* MTCC 3936 are summarized in following equations:

(2) Y1=+72.71−15.34A−15.12B+8.54C+11.62AB−3.36AC+2.25BC+0.11A2−22.01B2−5.79C2,Y2=+64.96−8.00A−11.42B+10.87C−2.14AB−0.90AC+1.69BC−4.50A2−17.23B2−7.58C2,Y3=+48.53−8.75A−9.59B+9.27C+5.08AB−0.64AC+1.78BC+5.44A2−13.24B2−2.11C2,Y4=+56.68−4.55A−6.86B+10.87C−1.57AB−3.81AC−2.05BC−3.23A2−16.34B2−6.17C2,Y5=+70.80−10.22A−10.15B+11.52C+4.55AB−1.98AC+1.26BC−1.92A2−22.28B2−9.17C2,

where *Y*_1_, *Y*_2_, *Y*_3_, *Y*_4_, and *Y*_5_ represent % decolorization of reactive yellow 18, reactive green 19, reactive red 31, reactive red 74, and reactive black 8, respectively; *A* = dye concentration, *B* = pH, *C* = incubation time, *AB* = dye concentration ∗ pH, *AC* = dye concentration ∗ incubation time, *BC* = pH ∗ incubation time, *A* ^2^ = dye concentration^2^, *B* ^2^ = pH^2^, and *C* ^2^ = incubation time^2^.

### 3.4. Statistical Analysis of the Results

Analysis of variance (ANOVA) was used to predict significance and accuracy of the CCRD models generated in the study (Table 3). Models were significant at a level of *P* value less than 0.05 in case of reactive yellow 18, reactive green 19, reactive red 74, and reactive black 8. However, in case of reactive red 31, model *F* value of 2.31 implies that model is not significant relative to noise. Model *F* value was 4.2 in reactive yellow 18, 4.5 in reactive green 19, 3.06 in reactive red 74, and 3.4 in reactive black 8. There was only a little chance that the model *F* could be this large due to noise. Dye concentration and pH are significant model terms in all the dyes except for reactive red 31. The *P* value denotes the significance of coefficients and is also important in understanding the pattern of mutual interactions between the variables. Similarly, *R* ^2^ values confirm that coefficient of determination (*R* ^2^) is highly reliable. It is also observed that in case of reactive yellow 18, both dye concentration as well as pH play significant role in dye decolorization by* C. militaris*. While the important response variable in case of dyes reactive red 74 and reactive green 19 is incubation time and in reactive black 18, it is pH. However, in case of reactive red 31, all the factors are acting independently and playing insignificant role in dye decolorization. It can be concluded from the findings that such studies prove very useful to predict influence of response variables on effective dye removal by* C. militaris* which was reported for the first time as a useful candidate for removal of synthetic dyes from textile industry effluents.

## 4. Discussion

Most of the previous studies have focused on* Phanerochaete chrysosporium* and* Trametes versicolor* for dye decolourization. There has been a growing interest in studying ability of a wide array of white rot fungi for use in various biotechnological applications. Hence, in the present research,* C. militaris* was explored for its color removal ability. The previous study by Kumar et al. [17] reveals that the rate of decolorization by white rot fungus* P. chrysosporium* decreases with increase in concentration from 20% to 100% for Amido Black-B dye solution. Similar observations were recorded during this study that with increase in concentration of the dyes, there was decrease in the dye decolorization rate.

Earlier, a study carried out by Young and Yu [18] demonstrates that general tendency of the fungal system is that higher concentrations of dye cause slower growth and require longer time periods for decolorization. Similar findings were observed when dye decolorization by* C. militaris* was assayed at different concentrations ranging from 200 to 250 ppm which were decolorized from 95 to 100% after 8–15 days of incubation, whereas the dyes at concentrations of 50 to 100 ppm were decolorized after 3–11 days of incubation. Cripps and his coworkers [19] suggested that* Phanerochaete chrysosporium* could remove 80–97% of Tropeolin O and Congo red within 5 days. According to the present study, reactive red 31 requires 6 days and reactive red 74 requires 11 days of incubation in liquid culture systems for 100% decolorization. Heinfling and his coworkers [20] reported that* Bjerkandera adusta* and* Trametes versicolor* remove 95% of HRB38 dye within 4 days.

Ligninolytic enzyme productions and dye decolorization by white rot fungi are both pH dependent and maximum activity was observed in the acidic range between 4 and 6 [21–23]. Results of this study led us to conclude that pH and incubation time both have significant effect on decolorization of dyes. This study also indicated potential of the statistical optimization tools and response surface methodology (RSM) that enables and helps in finding optimum levels of the most significant variables for color removal with minimum effort and time.

Application of white rot fungi in decolorization of synthetic textile dyes is well documented in literature (Table 4). Different species of white rot fungi are able to degrade various dyes at concentrations ranging from 20 to 1000 ppm (mg/L) using whole cells, spore suspension, and purified laccase enzyme preparations. This study is first of its kind involving use of culture supernatant of* C. militaris* containing crude enzyme mixture for degradation of synthetic dyes. A common mechanism of dye degradation has been proposed for white rot fungi which involve activity of their extracellular ligninolytic enzymes such as lignin peroxidases, manganese peroxidases, and laccases [24, 25]. Fungal extracellular peroxidases are nonspecific towards their substrate and thus can attack some recalcitrant chemicals of diverse structures, including organic-pollutants [26], individual azo, triphenylmethane, phthalocyanine, and heterocyclic dyes, as well as complex industrial effluents [25]. It has also been proved that the mechanism of oxidative cleavage of dyes and their decolorization is strain dependant [27]. A similar mechanism is being proposed here for decolorization/degradation of reactive dyes by* C. militaris* which seems to be dependent on structural stability of dyes also, when whole cells are employed for dye decolorization.

In case of reactive red 31, a peculiar pattern of dye adsorption and desorption was observed which might point out the structural instability or toxicity of the dye that results in maximum growth inhibition as observed in the present study. Other dyes like reactive yellow 18 and reactive red 74 are showing leaching of dye from fungal hyphae when they are added at higher concentrations to the growth media. Reactive dyes are anionic in nature [28] which makes covalent bond with the fiber. It is hypothesized that reactive dyes are first adsorbed on the fungal hyphae through passive forces involving interaction of anionic dyes with positively charged components of fungal cell wall, namely, chitin, glucans, and cellulose [29], where their oxidation or degradation by fungal enzymes takes place. When dyes are accumulated in excess on the surface of the fungal hyphae, they might start exhibiting toxic reactions like breakdown of the hyphae structure as indicated by growth inhibition that results in leaching of dyes back into the assay medium. Another possible reason could be the inconsistency in behavior of peroxidase enzyme expression in* Cordyceps* which was also reported earlier in a study involving use of* Schizophyllum commune* for decolorization of Solar brilliant red 80 where it seems to be growth dependent [25]. As the fungus enters in stationary phase, an inconsistent behavior of dye decolorization was observed which may be attributed to varying concentrations of lignin peroxidase, laccase, and Mn-peroxidase with respect to incubation time in the culture medium containing Solar brilliant red 80.

## 5. Conclusion

*Cordyceps militaris* has a great potential to decolorize synthetic textile dyes present in the effluents of textile industries and further reactor scale studies are required for actual industrial applications. Statistical optimization of conditions for the decolorization of synthetic dyes had been proved to be a valuable tool for the assessment of properties of* C. militaris* decolorization potential. These experimental designs can convert the process variable correlations into mathematical model that predicts where the response is likely to be identified. The optimized conditions for dye decolorization are identified as dye concentration 300 ppm and incubation time 48 h at pH 5.5 to achieve maximum dye decolorization. The model validation proved a good consistency between the experimental results and predicted response. Further work needs to be carried out to explore potential of the fungus in batch or continuous systems of bioremediation or as a consortium member for activated sludge process. Moreover, a detailed molecular study on functional characterization of* Cordyceps* peroxidases needs to be carried out to answer queries like the differential response of organism to various reactive dyes and the phenomenon of frequent dye leaching by fungal hyphae.

## Figures and Tables

**Table 1:** Preliminary analysis of dye decolorization capacity and wet weights of *Cordyceps militaris* MTCC 3936 in culture based systems.

% dye decolorization Concentration of dye used (ppm)																				
Incubation time (days)	Reactive green 19				Reactive yellow 18				Reactive red 31				Reactive red 74				Reactive rlack 8			
	50	100	200	250	50	100	200	250	50	100	200	250	50	100	200	250	50	100	200	250
0	0	0	0	0	0	0	0	0	0	0	0	0	0	0	0	0	0	0	0	0
1	63.72	38.95	21.64	19.06	81.81	63.15	49.12	48.27	48.78	17.98	72.25	23.79	57.67	28.57	19.66	9.87	23.26	1.43	16.11	12.51
2	81.86	56.46	47.87	59.36	92.72	82.45	55.93	58.62	80.48	50.79	82.19	78.62	62.79	58.52	37.28	11.39	49.38	14.69	16.57	12.40
3	95.34	71.68	59.36	68.09	**100**	85.96	86.44	65.51	46.34	57.14	92.67	88.62	79.06	71.42	72.88	29.11	83.06	37.19	21.30	18.50
4	95.58	91.98	74.55	79.76	100	89.47	96.61	72.41	21.95	92.06	93.71	95.86	87.44	82.02	93.55	66.07	84.69	80.10	53.19	39.75
5	96.74	95.58	93.19	89.88	100	**100**	**100**	89.65	82.92	92.14	99.47	96.89	95.34	86.63	96.94	72.15	95.91	94.53	77.36	57.97
6	99.06	98.36	96.28	95.10	100	100	100	**100**	**100**	**100**	**100**	**100**	98.60	94.93	98.64	79.24	97.34	94.92	95.27	80.85
7	98.37	98.36	97.26	96.27	100	100	100	100	80.48	100	100	100	96.74	93.54	98.30	86.07	100	96.48	97.20	85.46
8	98.60	96.23	**97.43**	96.66	100	100	100	100	100	100	100	94.82	93.48	83.41	92.54	86.83	89.79	92.97	95.67	93.88
9	97.67	99.18	94.16	97.27	100	100	100	100	60.97	100	100	94.13	99.06	96.31	99.66	91.13	99.59	95.05	92.14	97.54
10	95.34	96.07	92.75	95.99	96.36	100	98.30	41.37	75.60	100	81.73	95.17	88.37	88.47	93.89	88.10	96.12	96.22	92.75	93.61
11	**100**	**100**	96.20	97.33	100	100	100	100	100	100	100	100	**100**	**100**	**100**	93.41	**100**	93.88	96.20	95.33
12	100	100	96.02	97.22	100	100	100	72.41	100	100	100	100	100	100	100	95.44	100	96.09	96.02	95.50
13	100	99.79	95.26	93.44	100	100	100	61.90	100	100	100	100	96.19	100	100	87.94	100	95.70	**96.60**	94.84
14	100	99.17	95.46	94.42	100	100	100	90.47	100	100	100	100	94.76	100	100	96.41	100	96.87	96.50	**95.50**
15	95.34	98.96	96.05	**97.25**	100	100	100	95.23	100	100	100	100	95.23	100	100	**97.17**	98.76	**97.13**	96.50	95.39

**Wet weight (g/100 mL) **	3.73	3.70	3.68	2.45	6.94	6.54	5.07	2.03	4.15	3.19	1.40	0.65	5.18	4.21	4.00	2.16	5.32	3.93	3.88	3.79

**Table 2:** Experimental responses of dye decolorization obtained during CCRD analysis using CFS of *Cordyceps militaris*.

Variable factors				% dye decolorization responses obtained									
Sr. number	Conc. of dye (ppm)	pH	Incubation time (h)	Reactive yellow 18		Reactive green 19		Reactive red 74		Reactive red 31		Reactive black 8	
				*E*	*p*	*E*	*p*	*E*	*p*	*E*	*p*	*E*	*p*
1	300	5.5	76	19.23	20.1	22.47	25.24	14.43	20.95	12.62	26.96	9.8	25.51
2	500	7	72	20.00	21.1	30.97	25.75	16.96	22.95	26.00	35.76	20.90	32.41
3	300	5.5	88	80.76	81.2	72.81	61.81	67.98	57.50	72.81	58.15	82.75	64.25
4	500	4	72	51.21	52.0	51.20	49.50	43.81	25.72	51.28	41.22	49.49	41.10
5	100	7	24	13.33	12.4	13.38	20.90	5.9	17.58	10.71	21	7.83	18.20
6	636.3	5.5	48	33.33	34.0	20.86	38.78	26.60	39.88	34.24	49.20	33.85	48.20
7	300	5.5	48	73.07	75.0	68.68	64.96	59.46	56.68	50.05	48.53	71.10	70.80
**8**	**300**	**5.5**	**48**	**73.07**	**72.0**	**65.36**	**64.96**	**55.37**	**56.68**	**49.11**	**48.53**	**68.59**	**70.80**
9	300	5.5	48	73.07	73.1	65.82	64.96	57.31	56.68	47	48.53	73.28	70.80
10	500	7	24	24.44	26.4	5.28	2.43	12.05	12.95	17.73	14.94	12.14	10.82
11	300	2.97	48	1.96	2.13	34.05	35.45	3.87	22.03	4.23	27.21	0.75	24.86
12	100	4	72	70.72	69.09	54.35	63.02	55.62	57.52	67.15	70.17	71.30	74.60
13	300	5.5	48	73.07	72.4	64.51	64.96	53.20	56.68	47.82	48.53	69.18	70.80
14	0	5.5	48	100	99.0	91.83	65.68	72.43	55.19	93.93	78.64	99.72	82.58
15	300	5.5	48	73.07	74.07	62.58	64.96	58.58	56.68	49.38	48.53	70.13	70.80
16	100	4	24	69.00	69.01	31.81	42.85	27.24	24.05	63.44	53.91	59.67	50.13
17	500	4	24	53.65	53.2	48.58	32.94	52.82	43.94	49.42	27.53	48.88	24.56
18	300	8.02	48	6.25	7.2	6.65	2.98	21.05	−1.06	18.24	−5.06	17.62	−9.29
19	300	5.5	48	73.07	73.01	61.38	64.96	55.50	56.68	47.74	48.53	72.07	70.80
20	100	7	72	33.33	32.4	26.38	47.84	21.94	42.83	22.27	44.39	21.42	47.72
**RSM model analysis (significant at Pvalue less than 0.05)**				**Significant**		**Significant**		**Significant**		**Nonsignificant**		**Significant**	

*E* is experimental value and *p* is predicted value.

**Table 3:** ANOVA analysis of CCRD models generated for various dyes.

Sr. number	Source	Sum of squares	Df	Mean square	*F* value	*P* value, prob > *F*
Reactive yellow 18						
1	Model	15815.32	9	1757.26	4.21	**0.0175 significant**
2	*A*: dye conc.	3213.33	1	3213.33	7.70	0.0196
3	*B*: pH	3123.77	1	3123.77	7.49	0.0210
4	*C*: incubation	995.52	1	995.52	2.39	0.1535 (nonsignificant)
5	*AB*	1079.27	1	1079.27	2.59	0.1389
6	*AC*	90.32	1	90.32	0.22	0.6518
7	*BC*	40.50	1	40.50	0.097	0.7618
8	*A* ^2^	14.52	1	14.52	0.0151	0.8267
9	*B* ^2^	1986.89	1	1986.09	6.91	0.0252
10	*C* ^2^	482.85	1	482.85	1.16	0.3074
11	Residual	4173.36	10	417.34		
12	Lack of fit	4173.36	5	834.61		
13	Pure error	0.000	5	0.000		
14	Cor. total	19988.67	19			
**R** ^2^ ** 0.8043, adequate precision 7.894**						

Reactive red 31						
1	Model	6933.16	9	770.35	2.31	**0.1041 not significant**
2	*A*: dye conc.	1046.11	1	1046.11	3.14	0.1069
3	*B*: pH	1256.93	1	1256.93	3.77	0.0809
4	*C*: incubation	1174.09	1	1174.09	3.52	0.0901
5	*AB*	206.45	1	206.45	0.62	0.4496
6	*AC*	3.30	1	3.30	9.904*E* − 003	0.9227
7	*BC*	25.42	1	25.42	0.076	0.7881
8	*A* ^2^	427.00	1	427.00	1.28	0.2842
9	*B* ^2^	2527.02	1	2527.02	7.58	0.0204
10	*C* ^2^	64.29	1	64.29	0.19	0.6699
11	Residual	3334.55	10	333.45		
12	Lack of fit	3327.71	5	665.54	486.70	<0.0001
13	Pure error	6.84	5	1.37	2.31	0.1041
14	Cor. total	10267.71	19			
**R** ^2^ ** 0.6752, adequate precision 6.482**						

Reactive red 74						
1	Model	6876.62	9	764.07	3.06	**0.0483 significant**
2	*A*: dye conc.	282.71	1	282.71	1.13	0.3127
3	*B*: pH	643.52	1	643.52	2.57	0.1398
4	*C*: incubation	1612.56	1	1612.56	6.45	0.0294
5	*AB*	19.84	1	19.84	0.079	0.7839
6	*AC*	116.28	1	116.28	0.46	0.5108
7	*BC*	33.78	1	33.78	0.14	0.7209
8	*A* ^2^	150.73	1	150.73	0.60	0.4555
9	*B* ^2^	3845.40	1	3845.40	15.38	0.0029
10	*C* ^2^	549.00	1	549.00	2.20	0.1692
11	Residual	2500.81	10	250.08		
12	Lack of fit	2473.93	5	494.79	92.03	<0.0001
13	Pure error	26.88	5	5.38		
14	Cor. total	9377.42	19			
**R** ^2^ ** 0.6752, adequate precision 5.239**						

Reactive green 19						
1	Model	9189.45	9	1021.05	4.52	**0.0137 significant**
2	*A*: dye conc.	873.91	1	873.91	3.87	0.0775
3	*B*: pH	1782.22	1	1782.22	7.89	0.0185
4	*C*: incubation	1614.99	1	1614.99	7.15	0.0233
5	*AB*	36.68	1	36.68	0.16	0.6954
6	*AC*	6.53	1	6.53	0.029	0.8683
7	*BC*	22.88	1	22.88	0.10	0.7568
8	*A* ^2^	291.80	1	291.80	1.29	0.2822
9	*B* ^2^	4276.32	1	4276.32	18.94	0.0014
10	*C* ^2^	827.47	1	827.47	3.66	0.0846
11	Residual	2258.35	10	225.83		
12	Lack of fit	2225.27	5	445.05	67.27	0.0001
13	Pure error	33.08	5	6.62		
14	Cor. total	11447.80	19			
**R** ^2^ ** 0.8027, adequate precision 6.461**						

Reactive black 8						
1	Model	12726.34	9	1414.04	3.44	**0.0337 significant**
2	*A*: dye conc.	1426.78	1	1426.78	3.47	0.0920
3	*B*: pH	1408.20	1	1408.20	3.43	0.0938
4	*C*: incubation	1811.25	1	1811.25	4.41	0.0621
5	*AB*	165.53	1	165.53	0.40	0.5398
6	*AC*	31.40	1	31.40	0.076	0.7878
7	*BC*	12.78	1	12.78	0.031	0.8635
8	*A* ^2^	52.88	1	52.88	0.13	0.7272
9	*B* ^2^	7153.88	1	7153.88	17.41	0.0019
10	*C* ^2^	1211.01	1	1211.01	2.95	0.1167
11	Residual	4108.02	10	410.80		
12	Lack of fit	4092.24	5	818.45	259.38	<0.0001
13	Pure error	15.78	5	3.16		
14	Cor. total	16834.36	19			
**R** ^2^ ** 0.7560, adequate precision 6.410**						

**Table 4:** Status of white rot fungi in decolorization of textile dyes.

Sr. number	White rot fungi	Dyes decolorized	System employed	References
1	*Anthracophyllum discolor *	Reactive orange 165	Complex pellet	[30]
2	*Cordyceps militaris *MTCC 3936	Reactive green 19, reactive yellow 18, reactive red 31, reactive red 74, and reactive black 8	Cell-free supernatant	This study
3	*Datronia *sp. KAP10039	Reactive blue 19 (RB 19) and reactive black 5 (RB5)	Fungal pellet	[31]
4	*Fomes fomentarius *	Solophenyl red 3BL	Laccase enzyme	[24]
5	*Ganoderma *sp.	Congo red, rhodamine 6G, and malachite green	Spore suspension	[32]
6	*G. australe, G. applanatum,*and* G. resinaceum *	Remazol brilliant blue R (RBBR), orange G, Novacron yellow S-3R, amaranth, erionyl blue (A-R), and terasil navy S-2GL	Whole cell inoculum	[33]
7	*L. edodes *AMRL 119*, L. edodes *AMRL 12,and* L. edodes *AMRL 122	Remazol brilliant blue R (RBBR), orange G, amaranth, erionyl blue (A-R), and terasil navy S-2GL	Whole cell inoculum	[33]
8	*Poria *sp.	Congo red, rhodamine 6G, and malachite green	Spore suspension	[32]
9	*Pleurotus ostreatus *	Azo blue and malachite green	Laccase enzyme, whole cells	[14, 34]
10	*P. ostreatus *IBL-02	Reactive dye 222	Spore suspension	[35]
11	*P. ostreatus *AMRL 135, *P. ostreatus *AMRL 136, *P. ostreatus *AMRL 137, *P. pulmonarius *AMRL 177,and* P. pulmonarius *AMRL 179	Remazol brilliant blue R (RBBR), orange G, Novacron yellow S-3R, amaranth, erionyl blue (A-R), and terasil navy S-2GL	Whole cell inoculum	[33]
12	*P. sajor-caju *	Navy Hexie, red GF, and blue B 133	Spore suspension	[36]
13	*Phanerochaete chrysosporium*and* P. chrysosporium *IBL-03	Navy Hexie, red GF, and blue B 133, Amido black 10B and reactive dye 222	Spore suspension, fungal mycelium	[32, 35, 37]
14	*S. commune *IBL-06	Solar brilliant red 80	Whole cell inoculum	[25]
15	*Trametes *sp.	Congo red, rhodamine 6G, and malachite green	Spore suspension	[32]
